# Factors associated with contraceptive use among married women with disabilities in Sidama National Regional State, Ethiopia: a case-control study

**DOI:** 10.7717/peerj.21408

**Published:** 2026-06-16

**Authors:** Zelalem Tenaw, Rekiku Fikre, Melese Siyoum

**Affiliations:** Department of Midwifery, College of Medicine and Health Sciences, Hawassa University, Hawassa, Ethiopia

**Keywords:** Contraceptive, Married women, Accessibility, Disability, Ethiopia, Associated factors

## Abstract

**Background:**

Accessibility and use of contraceptives among women with disabilities are crucial for preventing reproductive health problems, which results in a compounded burden of disability and additional reproductive health-related challenges, such as limited access to services, heightened vulnerability to reproductive health problems, and pervasive social stigma. However, factors associated with contraceptive use among married women with disabilities in Ethiopia are not well described. This study aimed to determine the factors associated with contraceptive use among married women with disabilities.

**Methods:**

A community-based case-control study design was conducted among 484 randomly selected married women with disabilities (323 controls/non-users and 161 cases/users) in the Central Sidama Zone from February 02 to March 17, 2025. The cases were married women with disabilities using contraceptive methods, while the controls were married women with disabilities not using contraceptive methods. Data were collected with face-to-face interviews using a structured questionnaire. A multivariable logistic regression analysis model was used to analyse the data. The adjusted odds ratio (AOR) with a 95% confidence interval (CI) was used to report the measures of association.

**Results:**

This study revealed that being a follower of the Muslim religion was associated with a lower odds ratio (AOR = 0.29; 95% CI [0.10–0.88]). Higher educational attainment increased odds of contraceptive use: primary education (AOR = 3.40; 95% CI [2.03–5.71]) and secondary or higher education (AOR = 4.47; 95% CI [1.94–10.31]). Good knowledge about contraceptives (AOR = 4.27; 95% CI [2.24–8.13]), a positive attitude towards contraceptives (AOR = 3.61; 95% CI [2.14–6.09]), and accessibility of contraceptive services (AOR = 3.26; 95% CI [1.90–5.57]) were also significantly associated with contraceptive use.

**Conclusions:**

Religion, educational status, knowledge of contraceptives, attitude towards contraceptive use, and accessibility of services were associated factors associated with contraceptive use among married women with disabilities. Therefore, interventions should focus on disability-inclusive education, engaging religious leaders, and improving service accessibility through outreach or home-based services.

## Introduction

Women with disabilities are those who experience long-term physical or mental impairments that limit their ability to perform daily activities. These disabilities may include conditions such as extremity paralysis, the use of a wheelchair, blindness, deafness, or diagnosed mental disabilities (Leavesley & Porter, 1982; World Health Organization, 2009; Bickenbach, 2011). Globally, over one billion people (approximately 15% of the population) live with some form of disability, with 75–80% residing in developing countries (Bickenbach, 2011; Zziwa et al., 2019). In Ethiopia, while the exact number of people with disabilities remains unclear, the World Health Organization estimates that approximately 17.6% of the population is living with disabilities (Bickenbach, 2011). In the Sidama National Regional State, the Labor and Social Affairs Office reports an estimated 645,622 people with disabilities, of whom 121,377 are of reproductive age. Among these, 36.7% are married (Tenaw, Gari & Gebretsadik, 2023a; Tenaw, Gari & Gebretsadik, 2023b).

Despite international commitments such as the Sustainable Development Goals and the United Nations Convention on the Rights of Persons with Disabilities (UNCRPD), women with disabilities remain disproportionately marginalized in reproductive health care (MacKay, 2006; World Health Organization, 2009). Article 9 of the UNCRPD obliges states to ensure accessibility of facilities and information (Andersona Paul, 2000; Mosher et al., 2017), encounter multiple barriers to health care access, including physical inaccessibility of facilities, communication barriers, negative social attitudes, and systemic or procedural limitations within health services (Shakespeare, 1996; Andersona Paul, 2000; Smith, Yousafzai & Kasonka, 2004; Karen et al., 2009; World Health Organization, 2009; Porat et al., 2012; Zainol et al., 2018; Crabb Caitlin & Heller, 2020).

Evidence suggests that women with disabilities are less likely to access contraceptive services compared to their non-disabled counterparts and more likely to rely on less effective contraceptive methods (Sullivan, 2025). Around 25% of individuals with disabilities believe that the current contraceptive delivery system is not accessible to them (Beyene, Munea & Fekadu, 2019). In Ethiopia, the average prevalence of contraceptive utilization among people with disabilities is 26%, with rates ranging from 18% in Gondar (Beyene, Munea & Fekadu, 2019) to 34% in Arba Minch (Yesgat et al., 2020). However, little is known about the associated factors of contraceptive service utilization among married women with disabilities, particularly in rural areas. Prior research has identified several factors associated with contraceptive use among people with disabilities, including employment status (Beyene, Munea & Fekadu, 2019), attitudes, marital status, age, education, type of disability (Yesgat et al., 2020) and religion (Alomair et al., 2020).

However, many of these studies are cross-sectional, urban-based, or focused on mixed populations, leaving an evidence gap regarding the factors associated with contraceptive use among married women with disabilities, particularly those living in rural areas. Such approaches may mask rural-specific barriers, including limited access to services, lower availability of sexual and reproductive health information, and stronger sociocultural constraints. This gap underscores the need for a targeted assessment in rural settings. Identifying factors associated with contraceptive use in this population is essential to designing inclusive family-planning programs that address both informational and structural barriers. In Sidama, where a sizeable number of people with disabilities are of reproductive age, and a substantial proportion are married, evidence on the drivers of contraceptive uptake among married women with disabilities is limited. This study, therefore, aims to identify factors associated with contraceptive use among married women with disabilities in the Central Sidama Zone, Ethiopia.

## Materials and Methods

### Study design and setting

A community-based unmatched case control study was carried out from February 02/2025, to March 17/2025. We preferred this design because formal matching could have introduced overmatching and limited our ability to examine the effects of important covariates. In addition, given the limited number of people with disabilities available for inclusion, an unmatched case-control design was more feasible. The study was conducted in randomly selected kebeles (small administrative units in Ethiopia) of the Dale and Wonsho districts and in the Yirgalem city administration, Central Zone, Sidama National Regional State, Ethiopia. Sidama National Regional State is a newly established regional state, officially structured in the year 2019/2020. It is located 275 km south of Addis Ababa, with Hawassa serving as the capital city. The Sidama region comprises 30 districts and eight city administrations. According to the 2017 population projection by the Ethiopian Central Statistics Agency, the total population is estimated at 3,668,304, of which 49.59% (1,819,176) are female (Ethiopia Central Stastics Agency, 2017). The 2017 population projections were used, as no updated official and methodologically comparable estimates are currently available. According to a report from the Sidama Region Labour and Social Affairs Office, an estimated 645,622 individuals are living with disabilities in the region. Of these, approximately 121,377 are believed to be within the reproductive age group, and 36.7% are reported to be married (Tenaw, Gari & Gebretsadik, 2023a; Tenaw, Gari & Gebretsadik, 2023b).

### Population

The study population comprised married women with long-term physical, sensory, or mental impairments that limit their ability to perform daily activities. Disabilities included extremity paralysis or physical handicap, wheelchair use, blindness in both eyes, deafness, and diagnosed mental disabilities (Leavesley & Porter, 1982; Bickenbach, 2011). Participants must have resided in the study area for at least six months.

### Case participants

The case participants were married women with disabilities who reported using contraceptive methods during the study period.

### Control participants

The control participants were married women with disabilities who reported not using any contraceptive methods during the study period.

### Inclusion criteria

Married women with physical or sensory disabilities who have lived in the selected kebeles and sub-cities of Dale and Wonsho districts and Yirgalem city administration, Central Sidama Zone, for at least six months. Disability status was determined based on self-report using a structured questionnaire aligned with standard measures of functional difficulty.

**Cases**: Using any contraceptive method during the study period.

**Controls**: Not using any contraceptive methods during the study period

### Exclusion criteria

• Women with diagnosed mental disabilities (due to challenges in obtaining informed consent and data reliability).
• Women experiencing serious illness during data collection.

### Sample size determination

The sample size was calculated using the OpenEpi software (Open-Source Epidemiologic Statistics for Public Health, version 3.1). We used the unmatched case-control study calculator with the following assumptions: a 95% confidence level, 80% power, an odds ratio of 1.8, a case-to-control ratio of 1:2, and an estimated 48.1% prevalence of access to contraceptive information among controls (Yesgat et al., 2020). The largest required sample size based on this primary exposure variable was 440 participants. To accommodate an anticipated 10% non-response rate, the final sample size was adjusted to 484 participants (161 cases and 323 controls).

### Sampling procedure

Initially, based on available resources, population density, and socio-demographic characteristics representative of the Sidama population, two districts and one city administration were purposively selected. From these, 30 kebeles and the study participants were then randomly selected. A community-based survey was then conducted to identify eligible participants (married women with disabilities) and registered 510 (169 cases and 341 controls) individuals from the selected kebeles. The sample size was proportionally allocated across the 30 selected kebeles based on this registration. From the registered participants, 484 individuals, comprising 323 controls and 161 cases, were selected through simple random sampling. Based on the inclusion criteria, the cases and controls were recruited. Two controls were selected from the neighbourhood of each case using a pre-determined procedure. Neighbourhood-based selection was used solely to ensure that cases and controls arose from the same underlying population, not for analytic matching.

### Variables

The outcome variable in this study was contraceptive use, defined as the practice of using methods or devices to prevent pregnancy. The primary exposure variable was service accessibility, assessed through questions related to access to contraceptive information (media exposure), distance to health facilities (in minutes on foot), availability of transportation, building accessibility, and husband permission. Covariates included age, knowledge, attitude, ethnicity, employment status, educational level, religion, type of disability, and place of residence.

### Operational definition

**Accessibility:** Accessibility was measured using five variables: Information about contraceptive, the requirement of husband’s permission, accessibility of the health facility building, distance from home to the health facility, and the availability of transportation. Participants who scored all five points across these variables were categorized as having access to contraceptive services. The accessibility variable was constructed by requiring all five items to be positive to reflect comprehensive access across key domains; partial fulfilment was considered insufficient for classifying a service as fully accessible. All items were given equal weight, as no prior evidence supported differential weighting. This cut-off was pre-specified based on conceptual considerations and informed by previous literature.

**Attitude:** Attitude towards contraceptive was assessed using seven items: belief that contraceptive affects health, agreement that pregnancies should be planned, agreement that pregnancies should be spaced by at least two years, perception that contraceptive affects sexual intercourse, belief that using contraceptive causes divine anger, perception that contraceptive methods cause infertility, and belief that women are more responsible for using modern contraceptive methods. Measured on a 5-point Likert scale. Responses were summed to generate a total score, with higher scores indicating a more favourable attitude; scores above the mean were classified as favourable. In this study, total scores ranged from 14 to 34. Negatively worded items were reverse-coded before analysis. Internal consistency of the scale was assessed using Cronbach’s alpha, which demonstrated acceptable reliability (*α* = 0.72).

**Knowledge:** Knowledge of contraceptive use was assessed using three items measuring basic awareness, including the ability to define contraception, identify and list common contraceptive methods, and recognize common side effects associated with their use. Each item was coded as 1 for a correct response and 0 for an incorrect or “don’t know” response. Item scores were summed to generate a total knowledge score ranging from 0 to 3. Participants who correctly answered at least two items were classified as having good basic knowledge, while those who answered one or none were classified as having poor knowledge. The questionnaire was adapted from previously published studies, reviewed by subject experts to ensure content validity, and pretested to assess clarity and comprehensibility.

### Data collection procedure and quality assurance

The data collection questionnaire was adapted from different literature (Central Statistical Agency (CSA) (Ethiopia) and ICF, 2016; Mekonnen et al., 2020; Yesgat et al., 2020). The tool was initially prepared in English, translated into Sidamu Afoo, and then back-translated into English to ensure consistency and preserve the original meaning. Prior to data collection, the questionnaire was pretested on 5% of the sample in a non-selected area, and necessary revisions were made based on feedback. Data were collected through face-to-face interviews conducted in local language (Sidamu Afoo) by ten midwives fluent in the language and experienced in data collection. To maintain the 1:2 case-control ratio, two controls were interviewed for each case to increase statistical power and improve the precision of the estimated associations. Both groups were assessed using the same questionnaires.

A three-day training session was provided to the data collectors by the principal investigator and co-researchers. The training covered the study objectives, interview techniques, ethical considerations, questionnaire content and procedures ensuring data quality. During data collection, the principal investigator closely supervised and monitored the process, resolving any challenges that arose. Moreover, the principal investigator conducted daily reviews of completed questionnaires to check for completeness and accuracy, ensuring high-quality data.

### Data management and analysis

The Kobo Collect (version 2021.3.4) application was used to collect the data. Following data collection, the data were imported into Stata version 16 for analysis using the “SSC install kobo2stata” command.  Data cleaning included identifying variable types and assessing distributions using frequencies for categorical variables and means with standard deviations for continuous variables.

The outcome variable was contraceptive service utilization. Initially, bivariable logistic regression analyses were performed to examine the association between each independent variable and contraceptive use. Variables with a *P*-value < 0.20 in bivariable analysis were considered for inclusion in the multivariable logistic regression model. The multivariable model was then used to identify factors independently associated with contraceptive use, adjusting for potential confounders. Adjusted odds ratios (AORs) with 95% confidence intervals (CIs) and *P*-values < 0.05 were used to determine the statistical significance and strength of associations. Model diagnostics, including checks for multicollinearity and goodness-of-fit, were conducted to ensure the validity of the final model.

### Ethical considerations

Ethical clearance was obtained from the Hawassa University institutional review board with approval number of Ref. No: IRB/376/16. A support letter was obtained from the College of Medicine and Health Sciences at Hawassa University and submitted to the Sidama Public Health Institute. Subsequently, the Sidama Public Health Institute issued a support letter to the selected Woredas and city administration, health offices, health centers, and kebeles administrations. Finally, after explaining the purpose and procedures of the study, written consent was obtained from the participants. The data collection tool included a ‘yes/no’ prompt, where data collectors indicated whether the participant agreed or declined to participate in the study.

## Results

### Socio-demographic characteristics of study participants

This study involved 480 married women with disabilities, including 160 cases and 320 controls. The overall response rate was 99.17% (99.07% of cases and 99.38% of controls). The mean age of cases was 33.9 years with a standard distribution of 6.93 and 32.1 years for controls with a standard deviation of 7.11 years. Regarding educational status, 55 (34.4%) of the cases and 231 (72.2%) of the controls were unable to read and write. Conversely, 80 (50.0%) of cases and 73 (22.8%) of controls had completed primary education (Table 1).

**Table 1:** Socio demographic characteristics of married women with disabilities in Sidama Region, Ethiopia, 2025 (*N* = 480).

**Variables**	**Category**	**Cases (*N* = 160)** **n (%)**	**Controls (*N* = 320)** **n (%)**	***P*-value**
Age in years	Mean (SD)	33.9 (6.93)	32.1 (7.11)	0.98
Residence	Rural	110 (68.8)	216 (67.5)	0.78
	Urban	50 (31.2)	104 (32.5)	
Religion	Protestant	122 (76.25)	261 (81.56)	0.03
	Orthodox	19 (11.87)	30 (9.38)	
	Muslim	6 (3.75)	20 (6.25)	
	Catholic	13 (8.13)	9 (2.81)	
Ethnic group	Sidama	151 (94.37)	305 (95.31)	0.66
	Others^a^	9 (5.63)	15 (4.39)	
Educational status	Unable to read and write	55 (34.38)	231 (72.19)	<0.001
	Primary	80 (50)	73 (22.81)	
	Secondary and above	25 (15.62)	16 (5)	
Employment	Unemployed	157 (98)	315 (98.4)	0.80
	Employed	3 (2)	5 (1.6)	
Types of disability	Vision disability	31 (19.38)	73 (22.82)	0.21
	Hearing disability	34 (21.25)	78 (24.38)	
	Extremity paralysis	50 (31.25)	106 (33.12)	
	Wheelchaired	45 (28.12)	63 (19.68)	

**Notes.**

a Amhara, Oromo, Guragie and Wolayta.

### Accessibility and contraceptive-related characteristics of participants

Among the cases, 153 (95.6%) women reported having ever received counselling about contraceptives, compared to 171 (53.4%) of the control women. Additionally, in most cases 150 (93.75%) and 230 (71.87%) of the control women had heard information about contraceptives. Contraceptive service is accessible to 60 (37.5%) of the cases and 51 (15.9%) of the control women (Table 2). The chi-square test (*P* < 0.001) showed a significant difference in accessibility and contraceptive related characteristics between cases and control groups.

**Table 2:** Accessibility and contraceptive characteristics of married women with disabilities in Sidama Region, Ethiopia, 2025 (*N* = 480).

**Variables**	**Category**	**Cases (*N* = 160)** **n (%)**	**Controls (*N* = 320)** **n (%)**	***P*-value**
Ever counselled about contraceptive	No	7 (4.38)	149 (46.56)	<0.001
	Yes	153 (95.62)	171 (53.44)	
Heard information about contraceptive	No	10 (6.25)	90 (28.13)	<0.001
	Yes	150 (93.75)	230 (71.87)	
Distance from home to health facility	≥ 30 min on foot	65 (40.62)	183 (57.19)	0.001
	<30 min on foot	95 (59.38)	137 (42.81)	
Transport availability	No	14 (8.75)	121 (37.81)	<0.001
	Yes	146 (91.25)	199 (62.19)	
Building accessibility	No	14 (8.75)	112 (35)	<0.001
	Yes	156 (91.25)	208 (65)	
Husband permission	No	12 (7.50)	158 (49.38)	<0.001
	Yes	148 (92.5)	162 (50.62)	
Overall service accessibility	No access	100 (62.5)	269 (84.1)	<0.001
	Have access	60 (37.5)	51 (15.9)	

### Contraceptive knowledge and attitude among married women with disabilities

**Figure 1:**
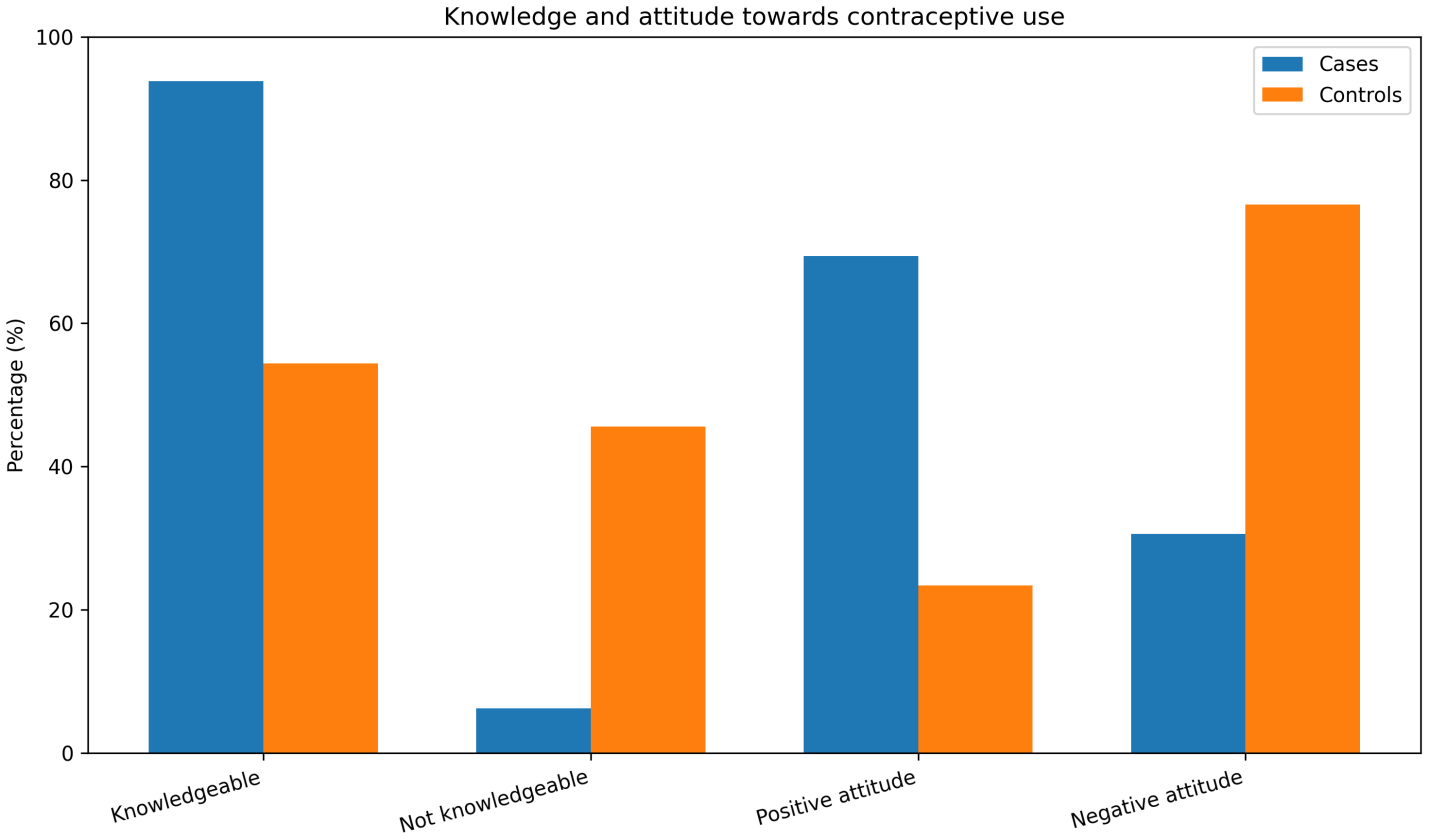
Knowledge and attitude towards contraceptive use of married women with disabilities in Sidama, Region, Ethiopia, 2025. Comparison the level of knowledge and attitudes towards contraceptive use between cases and controls. Knowledge was categorized as “knowledgeable” or “not knowledgeable” based on correct responses to standardized items assessing contraceptive awareness. Attitude was classified as either positive or negative using a validated Likert-scale questionnaire. Among the cases, 93.8% were knowledgeable, compared to 54.4% of the controls. Conversely, only 6.2% of cases were not knowledgeable, compared with 45.6% of controls. Regarding attitudes, 69.4% of cases demonstrated a positive attitude toward contraceptive use, compared with 23.4% of controls. On the other hand, 30.6% of cases and 76.6% of controls exhibited a negative attitude.

About 150 (93.80%) of the cases and 174 (54.4%) of the control women were knowledgeable about contraceptives. Additionally, 111 (69.4%) of the case women and 75 (23.4%) of the control women had a positive attitude towards contraceptives (Fig. 1). The chi-square test (*P* < 0.001) showed a significant difference in attitude and knowledge between cases and control groups regarding contraceptive.

### Factors associated with contraceptive methods use among married women with disabilities

In the bivariable logistic regression, age, religion, educational status, types of disability, contraceptive knowledge, attitude towards contraceptives and service accessibility were eligible (*P*-value ≤ 0.25) for inclusion in the multivariable logistic regression model. Except for types of disability, all the aforementioned variables were significantly associated with contraceptive use among married women with disabilities. However, in the multivariable logistic regression analysis, religion, educational status, knowledge, attitude and service accessibility were identified as the only significant factors associated with contraceptive use among married women with disabilities. We have assessed collinearity among the independent variables using the variance inflation factor (VIF) and tolerance values, and no significant multicollinearity was detected; the minimum VIF is 1.09, and the maximum is 1.6. Additionally, the predictive power of the regression model was evaluated using the Hosmer–Lemeshow test (*P* = 0.085), and the results indicate that the model has adequate fit and predictive ability.

Muslim married women with disabilities had 71% (AOR = 0.29; 95% CI [0.10–0.88]) lower odds of contraceptive use compared to Protestant married women with disabilities. The odds of contraceptive use increased threefold (AOR = 3.4; 95% CI [2.03–5.71]) for married women with disabilities who had a primary education and fourfold (AOR = 4.47; 95% CI [1.94–10.31]) for those with secondary or higher education, compared to married women with disabilities who were not educated. Furthermore, those who were knowledgeable about contraceptives had a fourfold (AOR = 4.27; 95% CI [2.24–8.13]) higher odds of contraceptive use compared to those who were not knowledgeable.

Additionally, married women with disabilities who had a positive attitude towards contraceptives were almost four times (AOR = 3.61; 95% CI [2.14–6.09]) more likely to use contraceptives than those with a negative attitude. Service accessibility is also a significant associated factor with contraceptive use among married women with disabilities; those who had access to contraceptive services had threefold (AOR = 3.26, 95% CI [1.90–5.57]) higher odds of using contraceptives compared to those without access (Table 3).

**Table 3:** Factors associated with contraceptives use among married women with disabilities in Sidama Region, Ethiopia, 2025.

**Variables**	**Category**	**Cases**	**Controls**	COR (95% CI)	AOR (95% CI)
Age in years	15 to 24	19	18	Ref	Ref
	25 to 34	81	153	0.5 (0.25, 1.01)^*^	0.62 (0.26, 1.48)
	35 to 44	53	120	0.42 (0.20, 0.86)^*^	0.54 (0.22, 1.33)
	45 to 49	7	29	0.23 (0.08, 0.65)^*^	0.53 (0.15, 1.90)
Residence	Rural	110	216	Ref	
	Urban	50	104	1.01 (0.71, 1.60)	
Religion	Protestant	122	261	Ref	Ref
	Orthodox	19	30	1.34 (0.73, 2.50)	0.52 (0.24, 1.16)
	Muslim	6	20	0.64 (0.25, 1.64)	0.29 (0.10, 0.88)^**^
	Catholic	13	9	3.09 (1.29, 7.43)^*^	1.30 (0.46, 3.72)
Educational status	Unable to read and write	55	231	Ref	Ref
	Primary	80	73	4.6 (2.99, 7.09)^*^	3.40 (2.03, 5.71)^**^
	Secondary and above	25	16	6.56 (3.29, 13.12)^*^	4.47 (1.94, 10.31)^**^
Types of disability	Vision disability	31	73	Ref	Ref
	Hearing disability	34	78	1.03 (0.57, 1.84)	1.27 (0.61, 2.63)
	Extremity paralysis	50	106	1.11 (0.65, 1.90)	0.67 (0.34, 1.31)
	Wheelchaired	45	63	1.68 (0.95, 2.97)^*^	0.76 (0.36, 1.62)
knowledge	Not-knowledgeable	17	147	Ref	Ref
	Knowledgeable	143	173	7.15 (4.13, 12.37)^*^	4.27 (2.24, 8.13)^**^
Attitude	Negative	59	245	Ref	Ref
	Positive	101	75	5.59 (3.70, 8.45)^*^	3.61 (2.14, 6.09)^**^
Service accessibility	No access	100	269	Ref	Ref
	Have access	60	51	3.17 (2.04, 4.91)^*^	3.26 (1.90, 5.57)^**^

**Notes.**

* *P*-value < 0.2.

** *P*-value < 0.05.

AOR Adjusted odds ratio CI Confidence interval Ref Reference category

## Discussion

This study identified several important factors associated with contraceptive use among married women with disabilities in Sidama National Regional State. Muslim women had 71% lower odds of contraceptive use compared to protestant women (AOR = 0.29; 95% CI [0.10–0.88]), women who completed primary education being 3.4 times more likely to use contraceptives (AOR = 3.40; 95% CI [2.03–5.71]) and those with secondary or higher education nearly 4.5 times more likely (AOR = 4.47; 95% CI [1.94–10.31]) than uneducated women. Knowledge about contraception increased the odds of use more than fourfold (AOR = 4.27; 95% CI [2.24–8.13]), while a positive attitude toward contraception was associated with a 3.6-fold higher likelihood of utilization (AOR = 3.61; 95% CI [2.14–6.09]). Additionally, accessibility of contraceptive services tripled the odds of use (AOR = 3.26; 95% CI [1.90–5.57]).

This study revealed that religion is one of the factors associated with the use of contraceptive methods. Married women with disabilities who are Muslim have a lower chance of using contraceptive methods compared to their protestant counterparts. This difference might be attributed to varying religious doctrines; different religious followers have distinct beliefs about contraception (Pinter et al., 2016). Some followers thought contraceptives are unacceptable, believing that children are gifts from God and should be born in accordance with divine will (Sundararajan et al., 2019). In contrast, others believe contraceptive as a moral responsibility, emphasizing the importance of caring for and protecting the family by planning its size. Evidence indicates that Muslim women have a negative attitude towards contraceptive methods (Alomair et al., 2020) and large family size is encouraged by principle (Okezie & Ogbe, 2010), which might be associated with lower utilization of contraceptive methods. This underscores the profound influence of religious beliefs and socio-cultural norms on family planning decisions.

Compared to married women with disabilities who are unable to read and write, those with a primary or higher level of education have a higher probability of contraceptive use. This might be because education usually enhances women’s knowledge of contraceptive methods, which is linked to contraceptive use. This finding is consistent with a study conducted among married women in Ethiopia (Tiruneh et al., 2016). Furthermore, educated women understand the importance of family planning, which is why they tend to have a lower fertility rate and higher usage of contraceptive methods (Okezie & Ogbe, 2010). Education not only increases knowledge of contraceptive methods but also enhances women’s autonomy and decision-making power, facilitating negotiation with partners and overcoming cultural barriers. Educated women are better equipped to understand the health and economic benefits of family planning, leading to lower fertility rates and higher utilization of contraceptive methods.

Contraceptive knowledge is another key associated factor with contraceptive use among married women with disabilities. Women who are knowledgeable about contraceptives are more likely to use contraceptive methods compared to those who have no contraceptive knowledge. This finding is consistent with the studies conducted in Ethiopia (Asres et al., 2022; Tenaw, Gari & Gebretsadik, 2023a; Tenaw, Gari & Gebretsadik, 2023b), Uganda (Ayiga & Kigozi, 2016) and Nigeria (Olajide et al., 2014). A possible reason for this difference might be the ability of knowledge to raise awareness and help overcome various cultural and social barriers that may hinder the use of contraceptives (Beyene, Munea & Fekadu, 2019). Moreover, attitudes towards contraceptives play a significant role in the use of contraceptive methods. Married women with disabilities who have a positive attitude are more likely to use contraceptive methods compared to those with a negative attitude. This finding is supported by several studies, including research conducted in various parts of ethiopia (Getinet et al., 2014; Meskele & Mekonnen, 2014; Asres et al., 2022). The possible justifications for this disparity are that women with a positive attitude towards contraceptives might tend to show a greater acceptance (James-Hawkins & Broaddus, 2016; Jaiswal et al., 2021) of and increased use of contraceptive methods. Their positive attitude might also help women to cope with and reduce social judgments and stigma (Rehnström Loi et al., 2019), while promoting open communication with their partners and health care providers.

Accessibility to contraceptive services is also another significant associated factor with contraceptive use among married women with disabilities. Women who have access to this service are more likely to use contraceptives compared to those for whom this service is not accessible. This finding is supported by previous studies conducted in various regions of Ethiopia (Tsegay, Gebremariam & Haile, 2017; Tenaw, Gari & Gebretsadik, 2023a; Tenaw, Gari & Gebretsadik, 2023b; Anshebo et al., 2025). The justification for this disparity is evident, as women with disabilities often face challenges that hinder their access to services (Olajide et al., 2014; Anshebo et al., 2025). These challenges may include physical barriers such as the distance to the health facility, difficulties with transportation, and accessibility issues within the building. Additionally, women may face challenges in obtaining information due to their specific disabilities, limited access to media and the need to secure permission from their husbands.

Notable differences were observed between cases and controls in education, knowledge, and attitude. While these factors may represent genuine determinants of the outcome, they may also indicate residual confounding or differences in group selection inherent to the case-control design. Although multivariable adjustment was applied to control for measured confounders, the possibility of unmeasured or incompletely measured confounding cannot be excluded.

These findings could be useful for different stakeholders concerned with factors associated with reproductive health services use, specifically contraceptive use among women with disabilities. This study was conducted among married women with disabilities residing in both urban and rural areas. It notably included rural residents who were excluded from previous studies. The other strength of this study is its use of a case-control study design, which provides stronger insights than a cross-sectional study for identifying the factors associated with contraceptive use. This study has some limitations. Due to the purposive selection of study districts, exclusion of certain disability groups, and the case–control design, the findings may not be broadly generalizable to all married women with disabilities in the Sidama region, but may provide useful insights for similar contexts. In addition, the knowledge and attitude measures were locally adapted and not previously validated in this population, which may affect reliability and comparability. Cross-cultural and language adaptation may have introduced misinterpretation. The use of interviewer-administered questionnaires may have increased social desirability bias. And also, classification based on mean-score cut-offs is sample-dependent and may limit generalizability. Finally, as this is a case–control study, the estimated odds ratios represent measures of association and should not be interpreted as direct estimates of prevalence or risk. Therefore, the magnitude of these associations should be interpreted with caution.

## Conclusion and recommendations

The use of contraceptive methods among married women with disabilities was associated with religion, educational status, knowledge of contraceptives, attitude towards contraceptives and accessibility of contraceptive services. Therefore, developing contraceptive-related educational programs, addressing misconceptions, enhancing positive attitudes towards contraceptive use by conducting counselling and awareness campaigns, engaging religious leaders to provide information that aligns with contraceptive use and breaks barriers related to religion, and providing contraceptive services at home are crucial to enhance contraceptive use among married women with disabilities. Future research should consider longitudinal designs to better establish temporal relationships, include a broader range of disability types, and explore health system and provider-level determinants of service accessibility.

##  Supplemental Information

10.7717/peerj.21408/supp-1 Supplemental Information 1 Raw data

10.7717/peerj.21408/supp-2 Supplemental Information 2 STROBE Checklist

10.7717/peerj.21408/supp-3 Supplemental Information 3 Codebook

## Additional Information and Declarations

### Data Availability

The following information was supplied regarding data availability: The raw data are available in the Supplemental File.
