# Supplementary material for: Factors associated with contraceptive use among married women with disabilities in Sidama National Regional State, Ethiopia: a case-control study
Source: PeerJ. 2026 Jun 16;14:e21408. doi: 10.7717/peerj.21408 (PMC13281746; doi:10.7717/peerj.21408)
Supplement: Supplemental Information 2 [file peerj-14-21408-s002.doc]

STROBE Statement—Checklist of items that should be included in reports of **case-control studies**

|  | Item No | Recommendation | Addressed in manuscript |  |
| --- | --- | --- | --- | --- |
| **Title and abstract** | 1 | (a) Indicate the study’s design with a commonly used term in the title or the abstract | Title clearly mentions "case-control study" |  |
|  |  | (b) Provide in the abstract an informative and balanced summary of what was done and what was found | Abstract includes background, objective, methods, main results, and conclusions |  |
| Introduction | | |  |  |
| Background/rationale | 2 | Explain the scientific background and rationale for the investigation being reported | Pages 3–4: Provides detailed global, national, and regional context, highlighting barriers faced by women with disabilities and the knowledge gap |  |
| Objectives | 3 | State specific objectives, including any prespecified hypotheses | End of introduction: Objective explicitly stated to identify determinants of contraceptive use among married women with disabilities |  |
| Methods | | |  |  |
| Study design | 4 | Present key elements of study design early in the paper | Under Materials and Methods: Described as a community-based unmatched case-control study |  |
| Setting | 5 | Describe the setting, locations, and relevant dates, including periods of recruitment, exposure, follow-up, and data collection | Pages 5–6: Detailed description of Sidama region, study districts, timeline (Feb 2 – Mar 17, 2025) |  |
| Participants | 6 | (a) Give the eligibility criteria, and the sources and methods of case ascertainment and control selection. Give the rationale for the choice of cases and controls | Pages 6–7: Inclusion/exclusion criteria clearly stated; rationale for neighborhood control selection provided |  |
|  |  | (b) For matched studies, give matching criteria and the number of controls per case | Not applicable (study is unmatched) |  |
| Variables | 7 | Clearly define all outcomes, exposures, predictors, potential confounders, and effect modifiers. Give diagnostic criteria, if applicable | Page 8: Outcome variable defined (contraceptive use); exposures and covariates (e.g., service accessibility, knowledge, attitude) described in detail |  |
| Data sources/ measurement | 8* | For each variable of interest, give sources of data and details of methods of assessment (measurement). Describe comparability of assessment methods if there is more than one group | Pages 9–10: Data collection via structured interviews, translated questionnaire, and training of data collectors described. Comparable for both cases and controls |  |
| Bias | 9 | Describe any efforts to address potential sources of bias | Page 10: Pretesting, random sampling, neighborhood controls, and supervision mentioned to minimize selection and information bias |  |
| Study size | 10 | Explain how the study size was arrived at | Page 8: Sample size calculated using OpenEpi, with assumptions clearly stated |  |
| Quantitative variables | 11 | Explain how quantitative variables were handled in the analyses. If applicable, describe which groupings were chosen and why | Page 11: Continuous variables summarized with means/SD; logistic regression for categorical variables |  |
| Statistical methods | 12 | (a) Describe all statistical methods, including those used to control for confounding | Page 11: Multivariable logistic regression, adjusted odds ratios, and confidence intervals reported |  |
|  |  | (b) Describe any methods used to examine subgroups and interactions | Not explicitly conducted; only main effects described |  |
|  |  | (c) Explain how missing data were addressed | Not explicitly stated – should note if data were complete |  |
|  |  | (d) If applicable, explain how matching of cases and controls was addressed | Not applicable (unmatched study) |  |
|  |  | (e) Describe any sensitivity analyses | Not performed or mentioned |  |
| Results | | |  |  |
| Participants | 13* | (a) Report numbers of individuals at each stage of study—eg numbers potentially eligible, examined for eligibility, confirmed eligible, included in the study, completing follow-up, and analysed | Page 12: Final analyzed numbers reported (160 cases, 320 controls); response rate given (99.17%) |  |
|  |  | (b) Give reasons for non-participation at each stage | Not detailed, but response rate suggests minimal non-participation |  |
|  |  | (c) Consider use of a flow diagram | No flow diagram included in the manuscript. |  |
| Descriptive data | 14* | (a) Give characteristics of study participants (eg demographic, clinical, social) and information on exposures and potential confounders | Table 1: Demographic and socio-economic characteristics |  |
|  |  | (b) Indicate number of participants with missing data for each variable of interest | No data were missed |  |
| Outcome data | 15* | Report numbers in each exposure category, or summary measures of exposure | Tables 2 and Figure 1 present data on knowledge, attitude, and accessibility by case/control status |  |
| Main results | 16 | (a) Give unadjusted estimates and, if applicable, confounder-adjusted estimates and their precision (eg, 95% confidence interval). Make clear which confounders were adjusted for and why they were included | Table 3: Both crude and adjusted odds ratios with 95% CI provided |  |
|  |  | (b) Report category boundaries when continuous variables were categorized | Table 3 shows categorical cut-offs (e.g., education levels) |  |
|  |  | (c) If relevant, consider translating estimates of relative risk into absolute risk for a meaningful time period | Not applicable here (case-control design) |  |

| Other analyses | 17 | Report other analyses done—eg analyses of subgroups and interactions, and sensitivity analyses | None |
| --- | --- | --- | --- |
| Discussion | | |  |
| Key results | 18 | Summarise key results with reference to study objectives | Page 14: First paragraph of Discussion clearly summarizes main results |
| Limitations | 19 | Discuss limitations of the study, taking into account sources of potential bias or imprecision. Discuss both direction and magnitude of any potential bias | Pages 16–17: Limitations such as exclusion of women with mental disabilities and potential information bias addressed |
| Interpretation | 20 | Give a cautious overall interpretation of results considering objectives, limitations, multiplicity of analyses, results from similar studies, and other relevant evidence | Pages 14–16: Findings compared with other regional and international studies, with thoughtful interpretation |
| Generalisability | 21 | Discuss the generalisability (external validity) of the study results | Page 17: Generalizability to Sidama married women with disabilities, excluding mental disabilities |
| Other information | | |  |
| Funding | 22 | Give the source of funding and the role of the funders for the present study and, if applicable, for the original study on which the present article is based | Stated in the declaration, as the authors received no funding for this work |

*Give information separately for cases and controls.

**Note:** An Explanation and Elaboration article discusses each checklist item and gives methodological background and published examples of transparent reporting. The STROBE checklist is best used in conjunction with this article (freely available on the Web sites of PLoS Medicine at http://www.plosmedicine.org/, Annals of Internal Medicine at http://www.annals.org/, and Epidemiology at http://www.epidem.com/). Information on the STROBE Initiative is available at http://www.strobe-statement.org.
