# Supplementary material for: Factors associated with contraceptive use among married women with disabilities in Sidama National Regional State, Ethiopia: a case-control study
Source: PeerJ. 2026 Jun 16;14:e21408. doi: 10.7717/peerj.21408 (PMC13281746; doi:10.7717/peerj.21408)
Supplement: Supplemental Information 3 [file peerj-14-21408-s003.docx]

| **Variable name** | **Variable description** | **Code** | **Meaning** |
| --- | --- | --- | --- |
| Age | Age of study participants | 1 | 15 to 24 |
|  |  | 2 | 25 to 34 |
|  |  | 3 | 35 to 44 |
|  |  | 4 | 45 to 49 |
| Residency | Residency of study participants | 0 | Urban |
|  |  | 1 | Rural |
| Religion | Residency of study participants | 1 | Protestant |
|  |  | 2 | Orthodox |
|  |  | 3 | Muslim |
|  |  | 4 | Catholic |
| Educational status | Educational status of study participants | 0 | Unable to read and write |
|  |  | 1 | Primary |
|  |  | 2 | Secondary and above |
| Types of disability | Types of disability of study participants | 1 | Vision disability |
|  |  | 2 | Hearing disability |
|  |  | 3 | Extremity paralysis |
|  |  | 4 | Wheelchaired |
| knowledge | Knowledge of study participants | 0 | Not-knowledgeable |
|  |  | 1 | Knowledgeable |
| Attitude | Attitude of study participants | 0 | Negative |
|  |  | 1 | Positive |
| Service accessibility | Service accessibility of study participants | 0 | No access |
|  |  | 1 | Have access |
| Employment | Employment of study participants | 0 | Unemployed |
|  |  | 1 | Employed |
| Ethnic group | Ethnic group of study participants | 1 | Sidama |
|  |  | 2 | Others◆ |
